# Supplementary material for: Loss of developmental diapause as prerequisite for social evolution in bees
Source: Biol Lett. 2019 Aug 14;15(8):20190398. doi: 10.1098/rsbl.2019.0398 (PMC6731480; doi:10.1098/rsbl.2019.0398)
Supplement: Figures S1 - S3 [file rsbl20190398supp1.pdf]

Santos, PKF; Arias, MC; Kapheim, KM. (2019) Loss of developmental diapause as prerequisite for social evolution in bees - published in *Biology Letters*

Supplementary Figures:

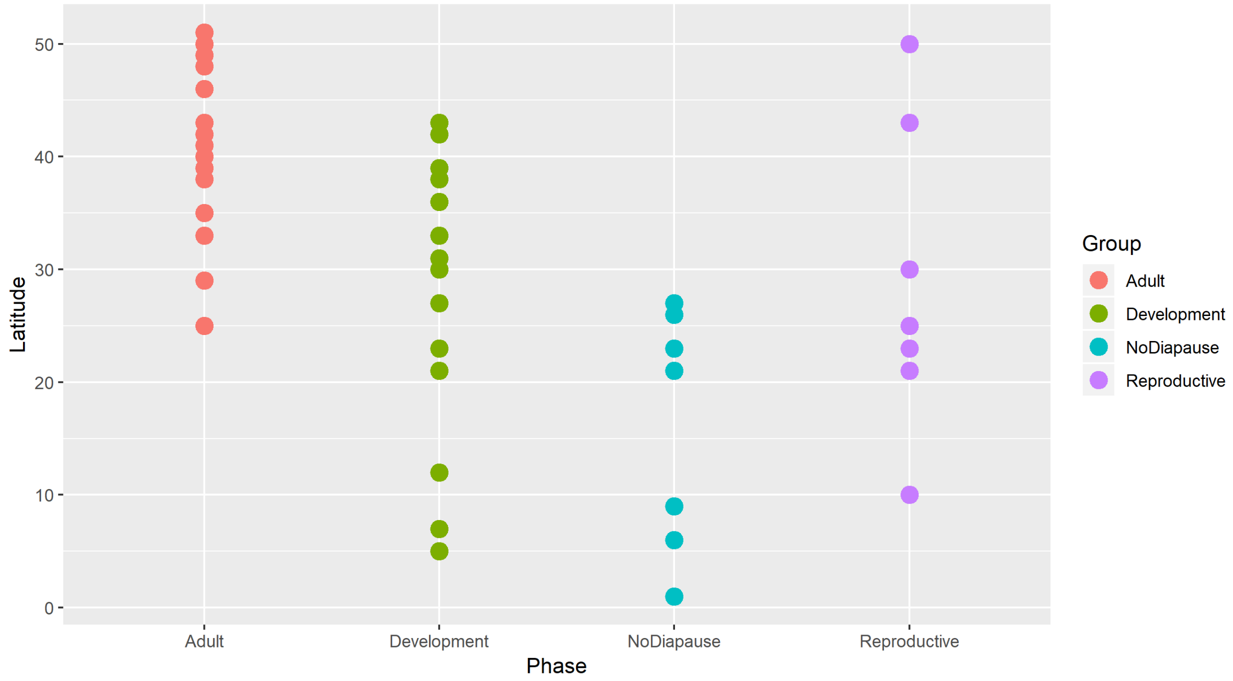

Figure S1: The relationship between diapause phase and latitude degrees. Species presenting adult diapause are more frequent at high latitudes and those that do not diapause are more frequent at low latitudes. Each dot represents a single species.

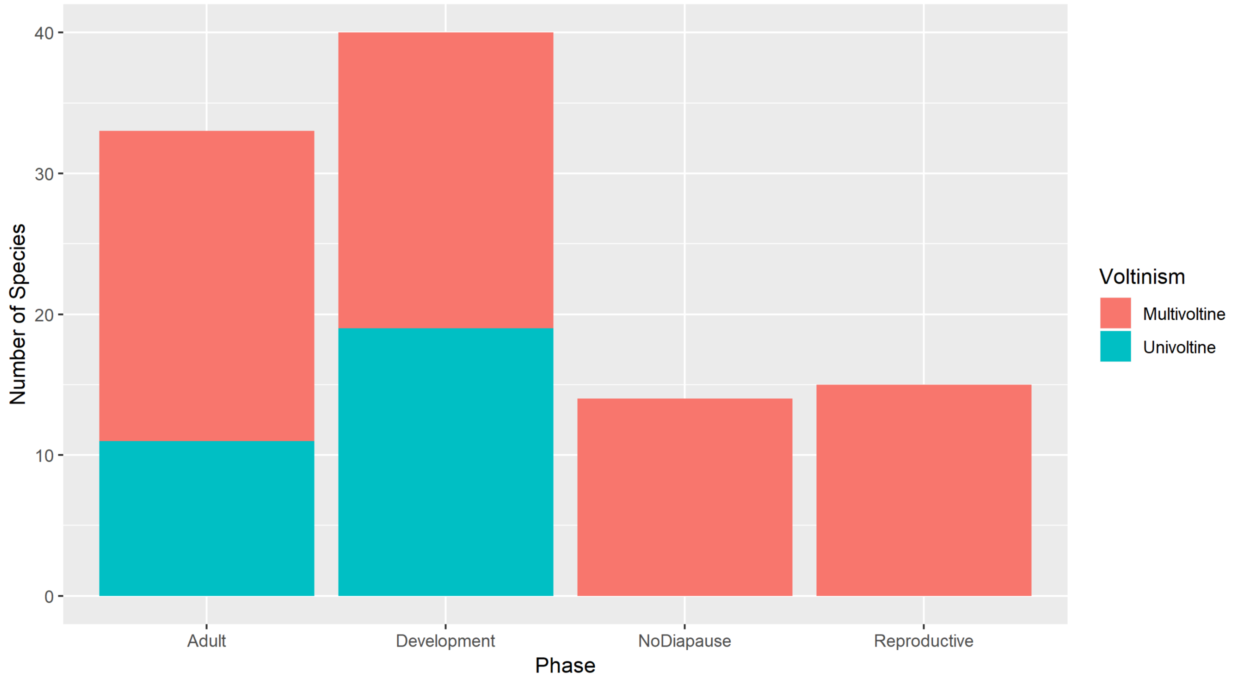

Figure S2: The relationship between diapause phase and voltinism.

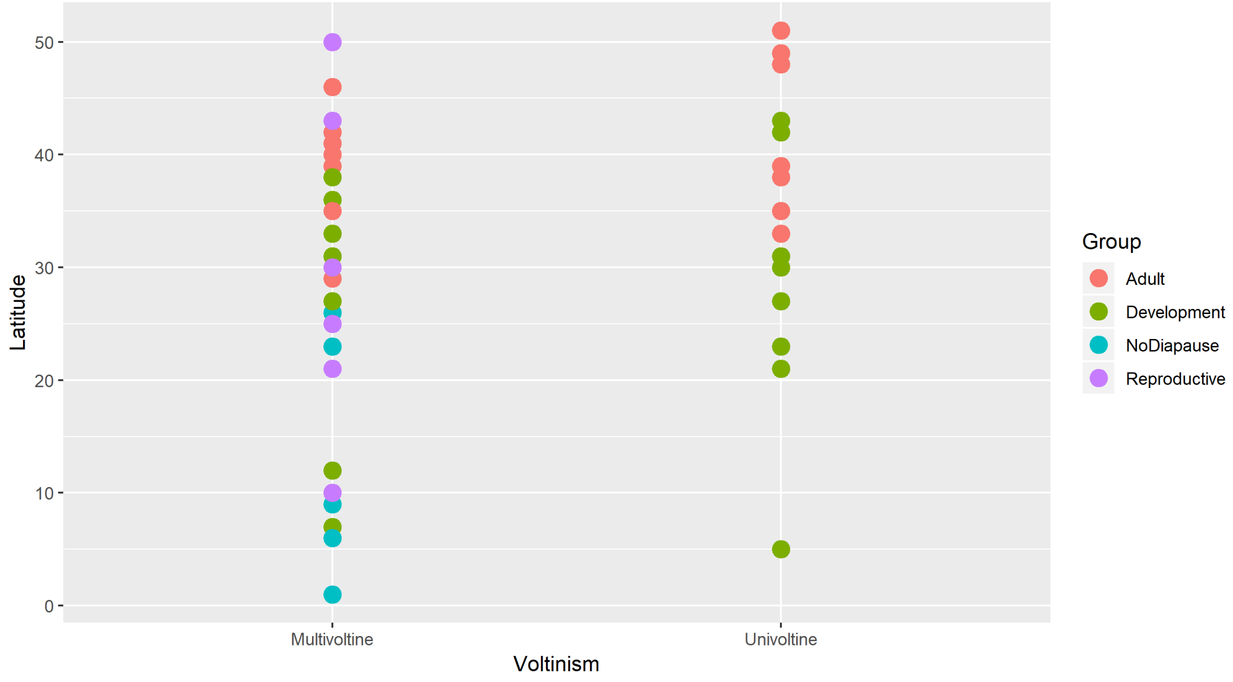

Figure S3: The relationship between voltinism and latitude degrees. Multivoltine species that do not diapause are all below latitude 30º. Univoltine species have developmental or adult diapause, and univoltine species that diapause as adults are all above a latitude 30º. Each dot represents a single species.
